# Electrical short-circuit in β-cells from a patient with non-insulinoma pancreatogenous hypoglycemic syndrome (NIPHS): a case report

**DOI:** 10.1186/1752-1947-4-315

**Published:** 2010-09-23

**Authors:** Robert Bränström, Erik Berglund, Pontus Curman, Lars Forsberg, Anders Höög, Lars Grimelius, Per-Olof Berggren, Per Mattsson, Per Hellman, Lisa Juntti-Berggren

**Affiliations:** 1 The Rolf Luft Research Center for Diabetes and Endocrinology, Department of Molecular Medicine and Surgery, Karolinska Institutet, Stockholm, Sweden; 2 Section of Endocrine Surgery, Department of Molecular Medicine and Surgery, Karolinska Institutet, Stockholm, Sweden; 3 Department of Oncology-Pathology, Karolinska Institutet, Stockholm, Sweden; 4 Department of Internal Medicine, Södersjukhuset, Stockholm, Sweden; 5 Department of Genetics, Pathology and Surgery, Uppsala University, Uppsala, Sweden

## Abstract

**Introduction:**

Non-insulinoma pancreatogenous hypoglycemic syndrome is a rare disorder among adults, and, to our knowledge, only about 40 cases have been reported in the literature.

****Case presentation**:**

The patient is a previously healthy 35-year-old Caucasian man. His symptoms began four years ago when he suddenly felt weakness in his legs and started sweating for unknown reasons. The symptoms worsened, and laboratory tests revealed hypoglycemia and hyperinsulinemia at the time of the symptoms. All diagnostics attempts using magnetic resonance imaging, computed tomography, and endoscopic ultrasound did not reveal any abnormalities. At this stage, surgical intervention was planned, and a distal 80% pancreatectomy was performed. The histopathologic and immunohistochemical investigations of the pancreas showed an increased number of islets of different sizes, more or less evenly distributed in the gland, but no insulinoma. Patch-clamp recordings from isolated pancreatic β-cells showed that, even at a low glucose concentration (3 mmol/L), the β-cell membrane was depolarized, and action potentials were seen. Surprisingly, in patch-clamp experiments, the addition of diazoxide had a marked effect on K-ATP channel activity and membrane potential, but no effect on insulin levels *in vivo *before surgery.

**Conclusion:**

This case report adds new information on the pathogenesis of non-insulinoma pancreatogenous hypoglycemic syndrome, as we performed an electrophysiologic characterization of isolated islet cells. We show, for the first time, that β-cells isolated from a non-insulinoma pancreatogenous hypoglycemic syndrome patient are constantly depolarized, even at low glucose levels, but display normal K-ATP channel physiology.

## Introduction

Nesidioblastosis is a rare, but well-recognized disorder of persistent hyperinsulinemic hypoglycemia in infancy. Nesideroblastosis is associated with mutations in adenosine triphosphate (ATP)-sensitive K^+ ^(K-ATP) channel subunits Kir6.2 and sulphonylurea receptor type 2 (SUR2). In adults, the most common cause of hyperinsulinemic hypoglycemia is solitary or multiple insulinoma(s). Recently, non-insulinoma pancreatogenous hypoglycemia syndrome (NIPHS) was described in adults as a novel cause of hyperinsulinism. NIPHS is often referred to as nesidioblastosis, even though NIPHS originates independent of mutations in K-ATP channel genes (*Kir6*.2 and *SUR2*) [1]. NIPHS is a very rare disorder among adults, to our knowledge, and only about 40 cases have been reported in the literature. Clinical presentation is heterogeneous, and here we describe a case of NIPHS in a 35-year-old man. In addition, this case report adds new information on the pathogenesis of NIPHS, as we performed an electrophysiologic characterization of isolated islet cells. We show, for the first time, that β-cells isolated from an NIPHS patient are constantly depolarized, even at low glucose levels, but display normal K-ATP channel physiology. Furthermore, we demonstrated that, although diazoxide had an effect on K-ATP channel activity *in vitro *in isolated β-cells, no effect of the drug was noted *in vivo*.

## Case presentation

The patient is a 35-year-old previously healthy Caucasian man. His symptoms began four years ago when he suddenly felt weakness in his legs and started sweating for unknown reasons. He noticed that if he lay down and consumed food, the symptoms disappeared. In the beginning, he had similar episodes with intervals of one to several months. These episodes were related to mental or physical stress or both. Later the attacks were more frequent, which eventually made him seek medical attention. Laboratory tests revealed hypoglycemia and hyperinsulinemia at the time of the symptoms. However, magnetic resonance imaging (MRI), computed tomography (CT), abdominal ultrasound, gastroscopy, and octreotide scintigraphy did not reveal any abnormalities, and he received the diagnose "hypoglycemia of unknown cause."

**Figure 1:**
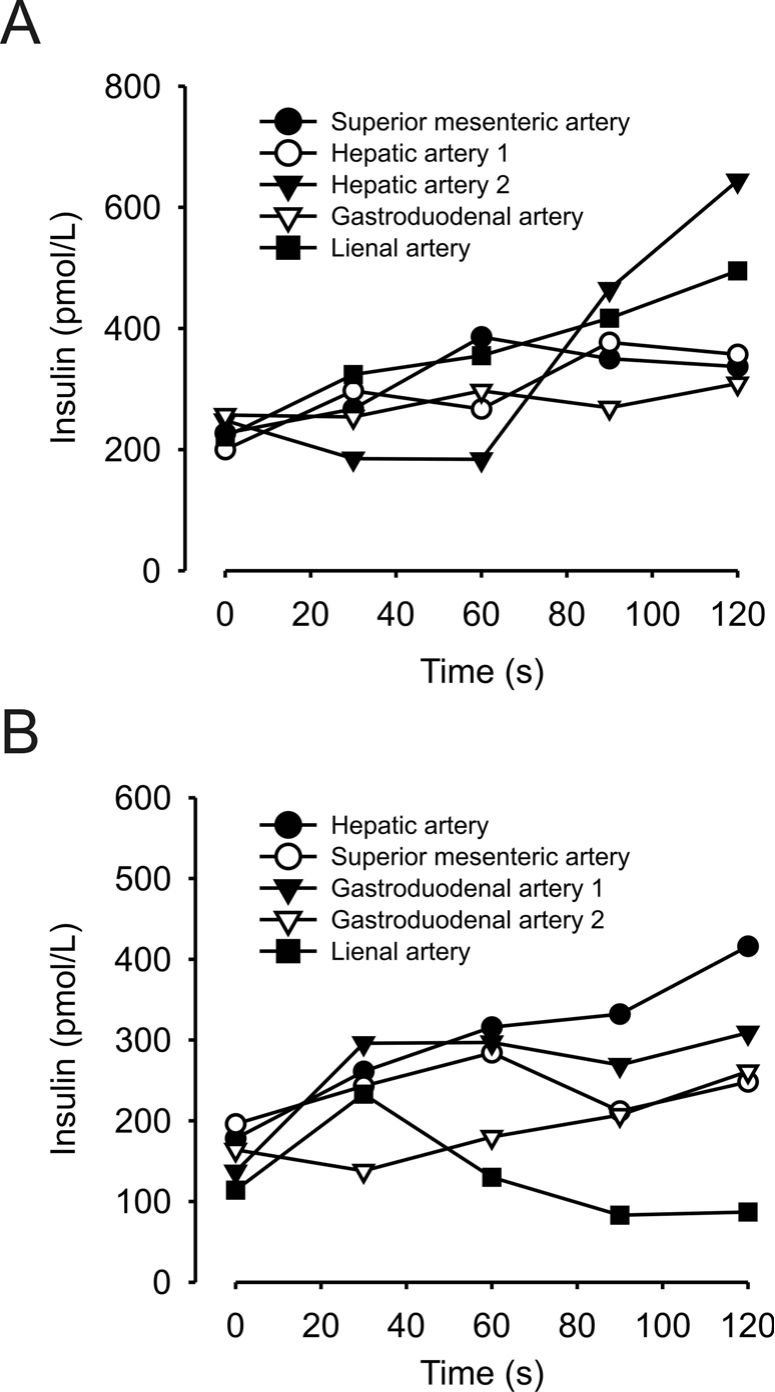
**Plasma insulin concentrations after selective intra-arterial calcium injections**. The investigation was done twice, with a month in between. Two injections were made in **(a) **the hepatic artery and **(b) **in the gastroduodenal artery.

The patient moved to Sweden three years after the onset of symptoms, which continued to become worse, leading to nearly daily and more severe attacks. He fainted several times and twice had traumatic injuries of the elbow and knee, necessitating orthopedic surgery. He was finally referred to the Karolinska University Hospital in Stockholm, where another careful investigation was performed. Again, repeated hypoglycemic attacks without relation to food intake were noted. P-glucose was as low as 1.4 mmol/L with simultaneously increased plasma insulin (470 pmol/L), plasma C-peptide (3 nmol/L), and plasma proinsulin (68 pmol/L) levels. Again, no signs of insulinoma were found with MRI, CT, endoscopic ultrasound, or positron emission tomography, using ^11^C-labeled 5-hydroxytryptophan as the tracer. Additional hormonal screening was normal, and analyses of insulin antibodies and sulphonylurea were negative. Two intra-arterial calcium stimulation tests [2] were performed (Figure 1), but no specific area of the pancreas demonstrating hypersecretion of insulin could be identified. Treatments with diazoxide, somatostatin, and cortisone were administered but did not prevent hypoglycemic attacks. The condition worsened, resulting in the need for food intake every hour, day and night, which, however, did not prevent the continuation of severe hypoglycemic episodes, leading in turn to a need for glucose infusions. At this stage, surgical intervention was decided on, by using a previous approach aiming for a subtotal pancreatectomy, leaving most of the pancreatic head.

**Figure 2:**
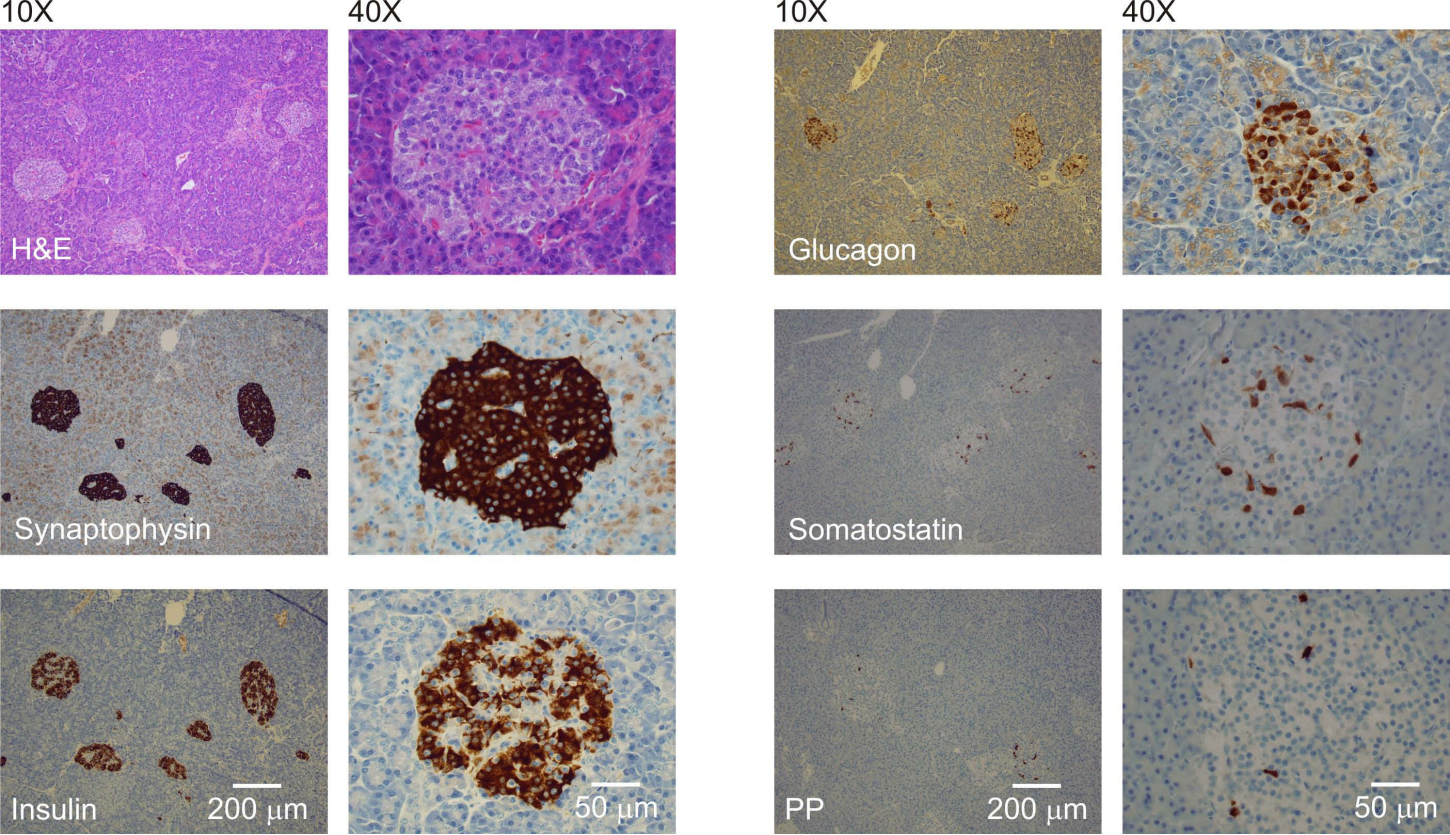
**Regular hematoxylin-eosin and immunohistochemistry stains using antibodies directed against synaptophysin, insulin, glucagon, somatostatin, and pancreatic polypeptide (PP)**.

No insulinoma could be detected during the operation, and a distal 80% pancreatectomy was performed. The histopathologic and immunohistochemical investigations of the pancreas showed an increased number of islets of different sizes more or less evenly distributed in the gland, no remarkably islet-cell hyperplasia in relation to ducts, and no insulinoma (Figure 2). A typical composition of endocrine cells was found, with a majority of insulin-secreting β-cells, fewer glucagon- and somatostatin-secreting cells, and only few pancreatic polypeptide-secreting cells. The nuclei of the β-cells were rather uniform and not enlarged, as had been reported in active hormone-producing β-cells.

**Figure 3:**
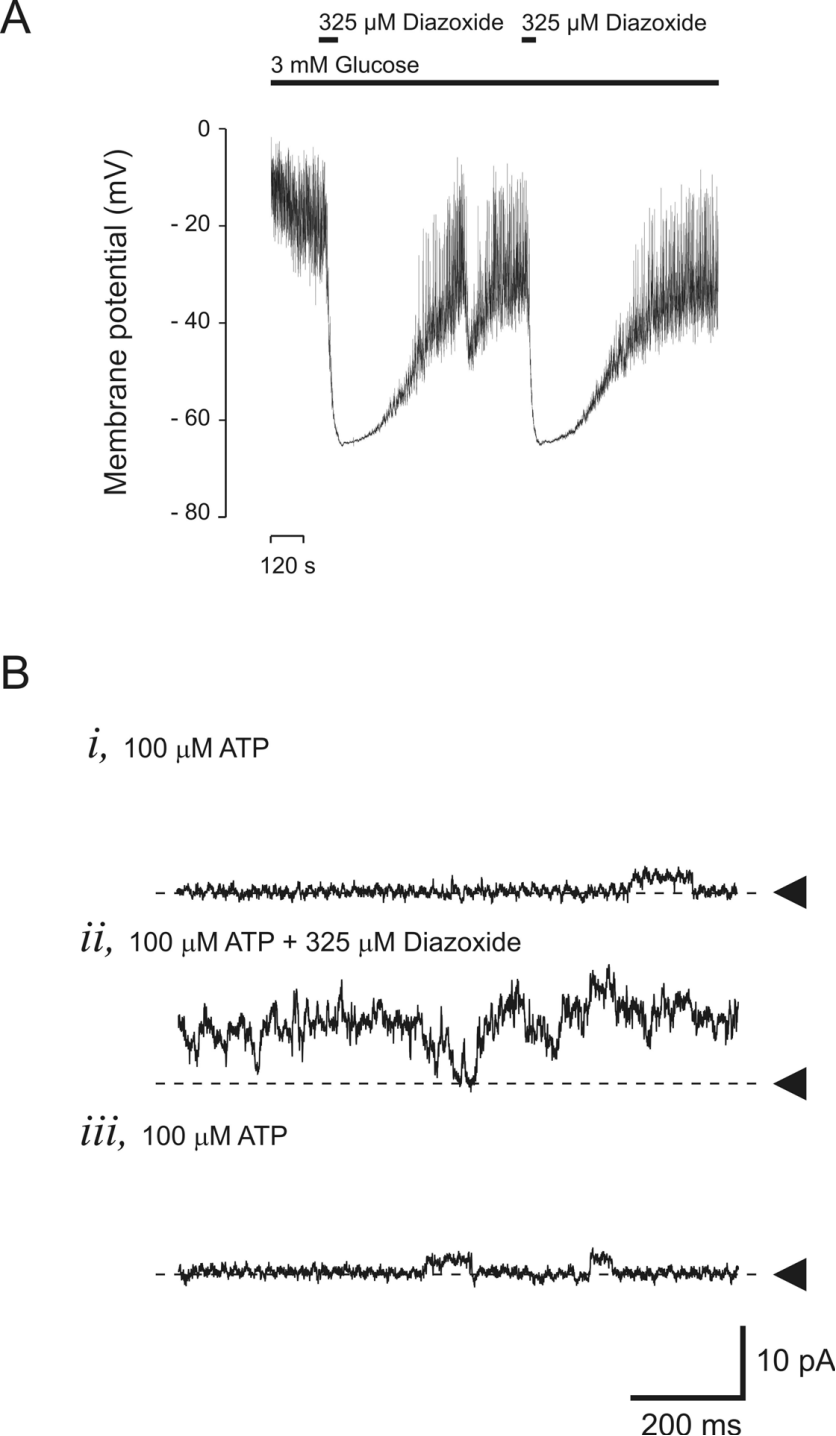
**Patch-clamp recording from pancreatic β-cells**. **(a) **In the presence of 3 mmol/L glucose, the β-cell was depolarized, but hyperpolarized after adding 325 μmol/L diazoxide. **(b) **K_ATP _channel recordings in isolated membrane patches. K_ATP _channel activity was blocked by 100 μmol/L ATP and activated by 325 μmol/L diazoxide. Pancreatic islets were isolated by a collagenase technique, and cell suspensions were prepared as previously described [8]. Experiments were performed on days two and three after isolation. Single-channel and whole-cell currents were recorded by means of the patch-clamp technique, by using an HEKA EPC-10 patch-clamp amplifier (HEKA Elektronik, Germany). Solutions and the set-up are the same as those described earlier by Bränström *et al. *[6]. In short, membrane potential recordings were made by the perforated-patch technique by using amphotericin dissolved in DMSO, and recordings were started when R_S _< 60 MΩ. All experiments were performed at room temperature (approximately 22°C).

We isolated islets and dispersed them into single cells, followed by analyses of single K_ATP _channel currents and membrane potentials by using the patch-clamp technique (Figure 3). Patch-clamp recordings from isolated pancreatic β-cells showed that, even at a low glucose concentration (3 mmol/L), the β-cell membrane was depolarized, and action potentials were seen (Figure 3a). The mean membrane potential in the presence of 3 mmol/L glucose was -38 ± 6 mV (measured between action potentials). In normal pancreatic β-cells, the membrane potential at 3 mmol/L glucose is between -60 and -70 mV. Diazoxide, a K_ATP _channel activator, hyperpolarized the membrane to -62 ± 8 mV (*n *= 3). In inside-out patches (Figure 3b), diazoxide was also able to reverse the blocking effect of ATP. The mean current decreased to 2.7 ± 1.2 pA in the presence of 100 μmol/L ATP, compared with 20 ± 9 pA before adding the nucleotide (*n *= 3; *P *< 0.01). In the continued presence of 100 μmol/L ATP, an addition of 325 μmol/L diazoxide increased the mean current to 7.8 ± 2.7 pA (*P *< 0.05). At the eight-month follow-up, the patient had neither hypoglycemia nor diabetes and had lost 13 kg of weight. Unfortunately, he has a memory-function disturbance, possibly due to repeated occasions of severe hypoglycemia.

## Discussion

Non-insulinoma pancreatogenous hypoglycemic syndrome (NIPHS) is a rare disorder among adults and, to our knowledge, only about 40 cases have been reported in the literature [1,3-5]. This case report adds new information, as we performed an electrophysiologic characterization of isolated islet cells. As the time window for experiments was limited (two to three days after isolation), we were able to estimate K_ATP _channel activity in response to only one concentration of ATP (100 μmol/L). Comparison of the blocking effect of 100 μmol/L ATP in these cells with that found in wild-type pancreatic β-cells [6] demonstrated a slightly decreased effect of ATP. Surprisingly, in patch-clamp experiments, addition of diazoxide had a marked effect on K_ATP _channel activity and membrane potential, but no effect on insulin levels *in vivo *before surgery.

One plausible reason for this discrepancy could be that the concentration of diazoxide was not high enough in the β-cells in the hyperplastic islets. Another possibility is that isolated β-cells, directly exposed to the drug, respond differently compared with cells in the natural milieu within the islets surrounded by other endocrine cells, as well as vessels and nerves.

Preoperative management of patients with hypoglycemia has been debated repeatedly [7]. Because of improvements in various imaging methods, the previous recommendations for using the surgeon's hand and intraoperative ultrasound as the most sensitive methods are abandoned more frequently now. Thus, even though they were not successful in the present patient, we recommend CT or MRI as well as endoscopic ultrasound as initial imaging procedures. If these are negative, we advocate intra-arterial calcium-stimulation testing, which is a sensitive technique [2]. However, in our patient, no specific area of the pancreas showed hypersecretion of insulin, which led to the decision to remove 80% of the pancreas. The histopathologic investigation, showing an increase number of islets evenly distributed within the resected part of the pancreas, was in agreement with the results from the intra-arterial calcium-stimulation tests. NIPHS may be surgically cured by a subtotal pancreatic resection if no intraoperative insulinomas are found. Surgery in the present patient was delayed because of diagnostic difficulties but also by emigration and immigration to Sweden. The resulting memory dysfunction in our patient underscores the importance of the fast handling of patients with hypoglycemic symptoms.

## Conclusion

This case report adds new information on the pathogenesis of NIPHS, as we performed an electrophysiologic characterization of isolated islet cells. We show, for the first time, that β-cells isolated from an NIPHS patient are constantly depolarized, even at low glucose levels, but display normal K-ATP channel physiology.

## Competing interests

All authors declare that no conflict of interest would prejudice the impartiality of this scientific work.

## Authors' contributions

All authors added significant contribution in all parts of the study, including study design, interpretation of the data, and conclusions. More specifically, RB, EB, LF, and POB handled the experimental parts of the study, including patch-clamp; AH and LG, the pathological evaluation; and PC, PM, PH, and LJB, the patient care and surgical management.

All authors read and approved the final manuscript.

## Consent

Written informed consent was obtained from the patient for publication of this case report and accompanying images. A copy of the written consent is available for review by the Editor-in-Chief of this journal.
